# Comparison of 2 shear wave elastography systems in reproducibility and accuracy using an elasticity phantom

**DOI:** 10.1097/MD.0000000000024921

**Published:** 2021-04-16

**Authors:** Jung Han Woo, Eun Young Ko, Boo-Kyung Han

**Affiliations:** Department of Radiology and Center for Imaging Science, Samsung Medical Center, Sungkyunkwan University School of Medicine, Seoul, Korea.

**Keywords:** breast ultrasound, elastography, shear wave elastography

## Abstract

This study aimed to compare the accuracy and inter- and intra-observer reproducibility of the measured elasticity between 2 shear wave elastography systems. Three breast radiologists examined 8 targets of 4 different levels of stiffness (size: 11 mm, 4 mm) in an elasticity phantom (Customized 049A Elasticity QA Phantom, CIRS, Norfolk, VA, USA) using 2 different shear wave ultrasound elastography systems: SuperSonic Imagine (SSI) (SSI, Aix en Provence, France) and ShearScan (RS-80A, Samsung Medison, Seoul, Korea). Three radiologists performed ultrasound (US) elastography examinations for the phantom lesions using 2 equipment over a 1-week interval. Intra- and inter-observer reproducibility and the accuracy of the measured elasticity were analyzed and compared between the 2 systems. The accuracy of shape was also analyzed by shape-matching between B-mode and elastography color image. Intra-class correlation coefficients (ICC) were used in statistical analysis. For measured elasticity, the intra-observer and inter-observer reproducibility were excellent in both SSI and ShearScan (0.994 and 0.998). The overall accuracy was excellent in both systems, but the accuracy in small lesions (4 mm target) was lower in SSI than ShearScan (0.780 vs 0.967). The accuracy of shape-matching on the elastography image was 59.0% and 81.4% in the SSI and ShearScan, respectively. In conclusion, the SSI and ShearScan showed excellent intra- and inter-observer reproducibility. The accuracy of the Young's modulus was high in both the SSI and ShearScan, but the SSI showed decreased accuracy in measurement of elasticity in small targets and poor shape-matching between the B-mode image and color-coded elastography image.

## 1 Introduction

Elastography is one of the notable advanced technologies in recent diagnostic ultrasound (US) systems.^[1]^ Recently, systems equipped with various methods that apply strain have become available. They include systems with strain elastography (SE), which requires manual compression vibration, and systems equipped with shear wave elastography (SWE) technology that supply vibration energy by means of ultrasound push.^[2]^ SWE systems provide quantitative information based on the local estimation of shear-wave propagation speed. SWE also provides a qualitative assessment of lesion and surrounding tissue, which is encoded in a color map superimposed on B-mode images.^[3–5]^ The results from previous reports have shown typical peri- or intra-tumoral stiffness in the color elastic map in some malignant lesions.^[5–9]^

Like other characteristics of US examination, US elastography results can vary according to the operators. With increasing types of elastography systems, there is not only operator-dependent, but also system-dependent variation.^[10–12]^ As a result, correlation and comparison of elastography results between the different systems is necessary. In clinical practice, we needed objective evidence that tracking breast lesions with different US elastography systems would make little difference in the measured elasticity representing benign or malignancy. There have been a few reports on the comparison of different elastography systems.^[10–12]^ However, they compared the results between systems with different elastography technologies such as SE and SWE, or even within the same SWE, compared the results of different acquisition methods of SWE such as point SWE and two-dimensional (2D) SWE.^[13–15]^

On this base, we needed to know whether we could follow up a lesion using SWE in different SWE systems of 2D methods without inter observer or intra observer differences Therefore, the purpose of our study was to compare inter- and intra-observer reproducibility in measuring quantitative elasticity and shape accuracy of color-mapping in 2 different systems using two-dimensional SWE. This is an original research through in vitro experimental study using elasticity phantom model.

## 2 Materials and methods

### 2.1 Elasticity phantom models

**Figure 1:**
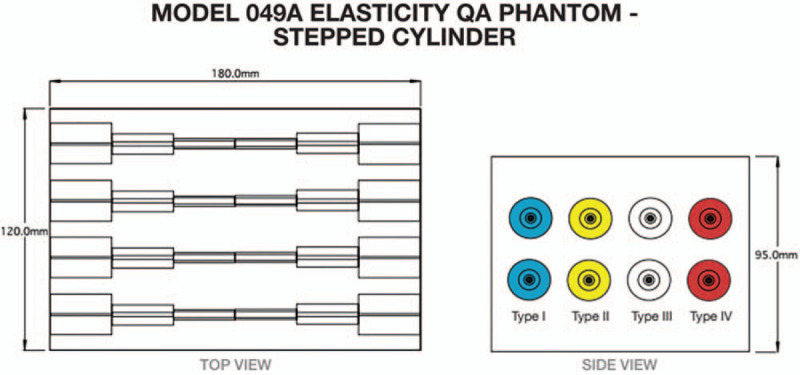
Top view and side view of the customized 049A Elasticity QA Phantom. Two sets of 4 stepped cylinders (from 18 mm–2 mm) with varying stiffness were embedded. The centers of the cylinders were uniformly located at an approximate depth of 2 cm.

For this study we used a commercially available Elasticity QA phantom model (Customized 049A Elasticity QA Phantom, CIRS, Norfolk, VA, USA) (Fig. 1). We used 2 areas of the stepped cylinders with diameter 11 mm and 4 mm. The characteristics of the lesions within phantom are summarized in Table 1.

**Table 1:** Characteristics of the lesions within phantom.

Lesion Type	Diameter (mm)	Lesion stiffness (kPa)	Background stiffness (kPa)
4	4	80 ± 10	2.6
	11		
3	4	48 ± 5	1.56
	11		
2	4	17 ± 4	0.55
	11		
1	4	11 ± 3	0.36
	11		

### 2.2 Data acquisition

**Figure 2:**
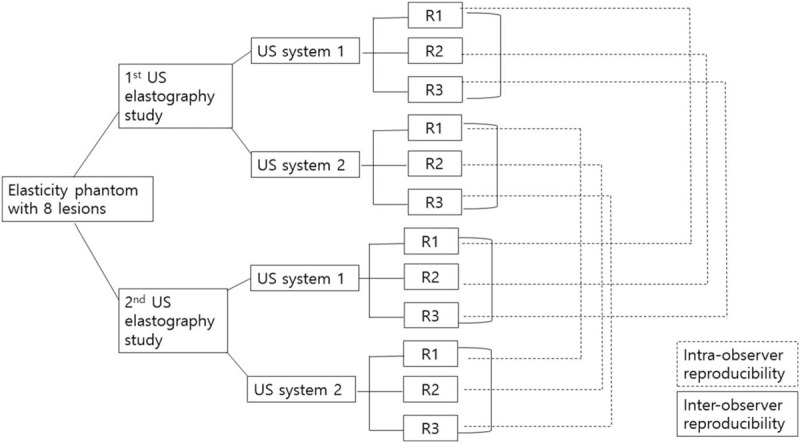
A flowchart explaining the process of study. Three radiologists (R1-3) measured elasticity of the phantom lesions using equipment 1 and repeated the same measurement using equipment 2 with 1 week interval.

**Figure 3:**
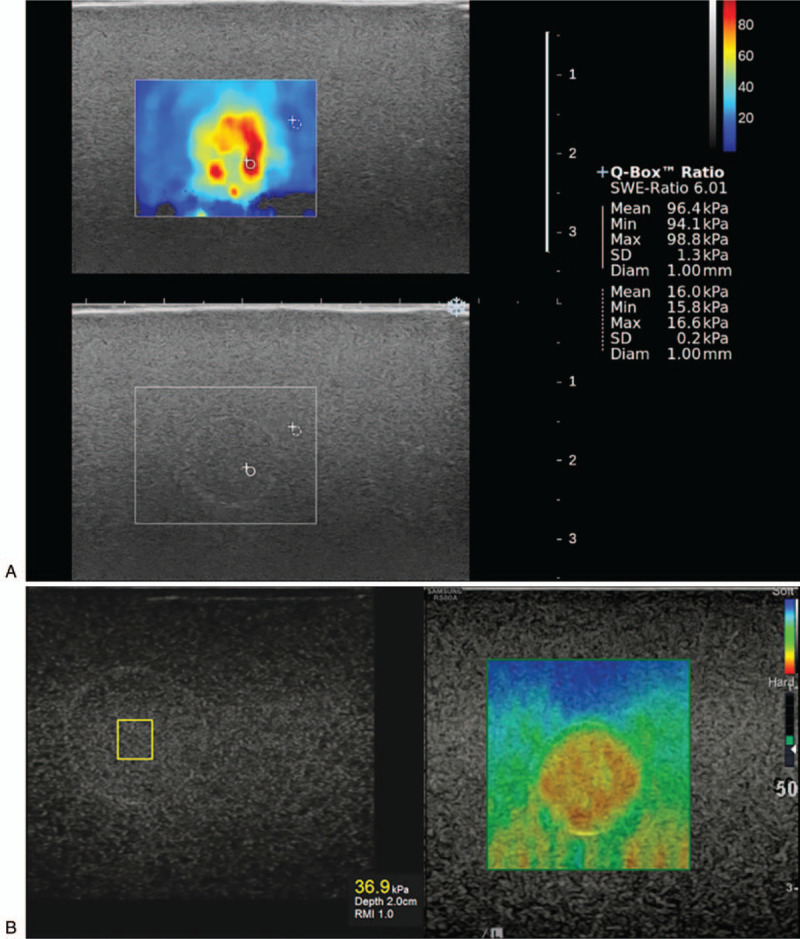
Measurement of elasticity by placing a region of interest (ROI) at the stiffest area within or just around the target using SuperSonic Imagine (A) and ShearScan (B).

Figure 2 explains how to proceed with our research. Three breast radiologists who had 15 to 25 years of experience in breast US and 5 years in elastography, examined 8 targets in the elasticity phantom: 11 mm and 4 mm size of targets with 4 different levels of stiffness. The phantom was imaged by 2 different SWE systems: SuperSonic Imagine (SSI) (SSI, Aix en Provence, France) and ShearScan (RS-80A, Samsung Medison, Seoul, Korea) using 50-mm 15 to 4 MHz linear array transducer. The B-mode image, color elstography image, and measurement of elasticity of 8 targets were obtained twice by each radiologist over a one-week interval. After scanning of the target with B-mode with 4 cm imaging depth, SWE was obtained. For measurement of elasticity, a region of interest (ROI) was placed at the stiffest area within or just around the target on a semitransparent color map of the tissue, ranging from blue (indicating the lowest stiffness) to red (indicating the highest stiffness) (0–100 kPa). Mean, minimum, and maximum values of elasticity were measured automatically following placing the ROI, and mean value was used to analyze the accuracy and compare the agreement. The elasticity ratio between the lesion and background was automatically calculated by placing another ROI at the representative area of the background (Fig. 3A). Color elastography and measurement of elasticity were separated in ShearScan (Fig. 3B). After measuring the elasticity, the elastic score was calibrated to 0 to 100 kPa scale based on the color of stiffness in the color elastography. To compare the reproducibility of the measurement, the same procedure was repeated with a week interval and results were compared to evaluate the conformity between 2 data sets.

### 2.3 Data analysis

**Figure 4:**
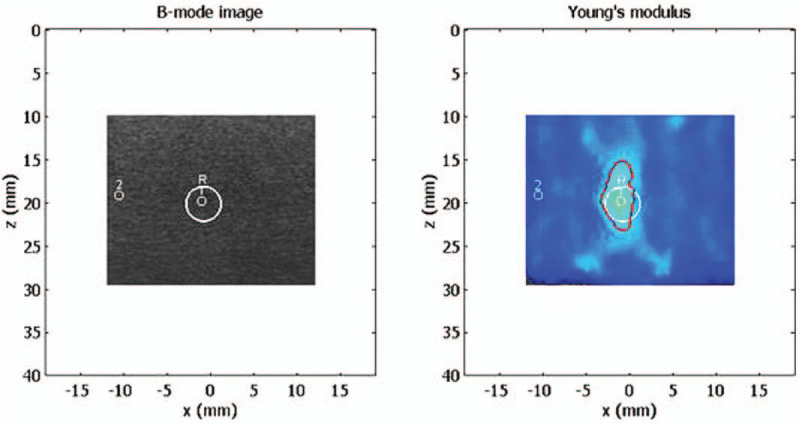
Measurement of shape accuracy. Shape of the lesion on the color elastography was automatically drawn by setting the threshold according to the known elasticity of the lesion. The real shape is drawn by automatic matching of the B-mode image with the color image of elastography after overlapping of 2 images together.

We analyzed the accuracy of the measured elasticity by comparing the measured results with the known elasticity of the lesions within the phantom. We evaluated intra- and inter-observer reproducibility of measuring elasticity and ratio of elasticity between the lesion and background. To evaluate the shape accuracy, B-mode images and paired elastography images of the lesion displayed on the same screen were used. The margin of the lesion on the color image of elastography was automatically drawn by setting the threshold according to the known elasticity of the lesion, and the real margin of the lesions on B-mode was overlaid on the color image of elastography (Fig. 4). Two margins of overlapped images should match as 1 line. The accuracy of shape was calculated using following formula:

Shape Accuracy=(1 - area of mismatch / area of the target)×100(%)

Because a small mismatched area in 1 examination could results in a substantial change in shape accuracy, we used 6 pairs of matched B-mode and color elastography images obtained by 3 radiologists and took the mean of 6 examinations for 1 lesion in comparing the shape accuracy.

### 2.4 Statistical analysis

Statistical analyses were performed using the SSPS software (IBM SPSS Statistics 21; IBM, Korea). Intra-class correlation coefficient (ICC) was used to compare the coherence of each variable for intra- and inter-observer reproducibility. For comparing the percentage accuracy of shape accuracy between the 2 elastography systems, a *t*-test was used. A *P* value <.05 was considered statistically significant.

Approval of an ethics committee or institutional review board was not necessary for this experimental study using phantom.

## 3 Results

The results of intra-observer reproducibility assessed by ICC in SSI and ShearScan are shown in Table 2. The intra-observer reproducibility was excellent in both systems. The measured intra-observer reproducibility was 0.990 to 0.996 in SSI and 0.996 to 0.999 in ShearScan for Young's modulus, and the elasticity ratio was 0.869 to 0.989 in SSI and 0.991 to 1.000 in ShearScan (*P* < .001). Measurements of elasticity in the 11 mm lesion showed slightly higher reproducibility than those in the 4 mm lesion, and were slightly higher in the ShearScan than in the SSI, but the difference was not significant.

**Table 2:** Intra-observer reproducibility: elasticity and ratio between the lesion and background.

	Elasticity			Ratio
	Overall (C.I.)	11 mm (C.I.)	4 mm (C.I.)	Overall (C.I.)
SSI				
R1	0.990 (0.954–0.998)	0.999 (0.979–1.000)	0.943 (0.376–0.996)	0.989 (0.944–0.998)
R2	0.996 (0.983–0.999)	0.997 (0.952–1.000)	0.989 (0.844–0.999)	0.970 (0.865–0.994)
R3	0.995 (0.977–0.999)	0.998 (0.964–1.000)	0.986 (0.806–0.999)	0.869 (0.512–0.972)
ShearScan				
R1	0.999 (0.994–1.000)	0.999 (0.981–1.000)	0.999 (0.988–1.000)	0.998 (0.991–1.000)
R2	0.996 (0.979–0.999)	0.997 (0.949–1.000)	0.995 (0.927–1.000)	0.991 (0.959–0.998)
R3	0.998 (0.991–1.000)	0.999 (0.981–1.000)	0.998 (0.973–1.000)	1.000 (0.999–1.000)

Inter-observer reproducibility was 0.991 to 1.000 and 0.996 to 1.000 in SSI and ShearScan for Young's modulus, 0.861 to 0.986 and 0.992 to 0.994 for ratio in the SSI and ShearScan (*P* < .001) (Table 3). Inter-observer reproducibility was excellent in both systems. ShearScan and 11 mm lesion showed slightly higher value than SSI and 4 mm lesion, but the difference was not significant.

**Table 3:** Inter-observer reproducibility of R1 vs R2 and R2 vs R3: elasticity and ratio between the lesion and background.

	Elasticity			Ratio
	Overall (C.I.)	11 mm (C.I.)	4 mm (C.I.)	Overall (C.I.)
SSI				
R1 vs R2				
1st	0.994 (0.970–0.999)	1.000 (0.996–1.000)	0.957 (0.490–0.997)	0.986 (0.932–0.997)
2nd	0.995 (0.977–0.999)	0.995 (0.926–1.000)	0.993 (0.902–1.000)	0.971 (0.864–0.994)
R2 vs R3				
1st	0.991 (0.957–0.998)	0.994 (0.916–1.000)	0.971 (0.633–0.998)	0.861 (0.456–0.971)
2nd	0.996 (0.981–0.999)	0.998 (0.975–1.000)	0.975 (0.677–0.998)	0.952 (0.781–0.990)
ShearScan				
R1 vs R2
1st	1.000 (0.999–1.000)	1.000 (0.995–1.000)	0.996 (0.936–1.000)	0.994 (0.971–0.999)
2nd	0.997 (0.986–0.999)	0.997 (0.962–1.000)	0.999 (0.979–1.000)	0.994 (0.972–0.999)
R2 vs R3				
1st	0.998 (0.992–1.000)	0.999 (0.977–1.000)	0.999 (0.982–1.000)	0.985 (0.926–0.997)
2nd	0.999 (0.997–1.000)	1.000 (0.995–1.000)	0.999 (0.988–1.000)	0.992 (0.959–0.998)

In both intra-observer and intra-observer reproducibility, the larger lesion and ShearScan showed slightly better results, but it was not significant because there was no statistical significance and all results were excellent even the lowest reproducibility of mearing elasticity was 0.990.

In terms of the accuracy of the measured elasticity, both systems showed excellent to good agreement between the measured elasticity and known elasticity of the lesions within the phantom (Table 4). In particular, the mean of the measured elasticity was accurate in the 11 mm lesion as ICC 0.972 with SSI and 0.955 with ShearScan (*P* < .05). However, the results for the 4 mm lesion were a little different, the mean ICC in the 4 mm lesion was 0.967 with ShearScan (*P* < .05) but 0.780 with SSI (*P* > .05). Measuring elasticity in a small lesion such as 4 mm with SSI was less accurate than in other cases, but was still good with mean ICC of 0.780.

**Table 4:** Accuracy of the measured elasticity of 3 observers compared to known values of phantom lesions.

	11 mm	4 mm
SSI		
R1	0.973 (C.I. 0.653–0.998)	0.800 (C.I. -0.263–0.986)
R2	0.966 (C.I. 0.578–0.998)	0.720 (C.I. -0.431–0.979)
R3	0.978 (C.I. 0.703–0.999)	0.821 (C.I. -0.206–0.987)
mean	0.972	0.780
ShearScan		
R1	0.950 (C.I. 0.436–0.997)	0.967 (C.I. 0.585–0.998)
R2	0.956 (C.I. 0.481–0.997)	0.967 (C.I. 0.588–0.998)
R3	0.966 (C.I. 0.575–0.998)	0.966 (C.I. 0.577–0.998)
mean	0.955	0.967

The results of shape accuracy, which means how well the color mapping of elastography matched with B-mode image of the lesion, are shown in Table 5. Overall, SSI showed a lower shape accuracy than ShearScan by showing 59.0% accuracy of shape-matching between B-mode and elastography images while ShearScan showed 81.4% accuracy of shape-matching between the 2 images (*P* < .05). The mean shape accuracy of the 11 mm lesion was 87.4% in ShearScan and 67.1% in SSI, but the difference between the 2 systems was not significant (*P* = .17). However, the results in the 4 mm lesion were 75.4% in ShearScan and 51.0% in SSI, and the difference was significant (*P* = .04). SSI showed significantly lower shape accuracy compared with ShearScan, especially in small lesions. There was no correlation between the accuracy of measuring elasticity and level of elasticity.

**Table 5:** The mean accuracy of shape-matching between B-mode and elastography images.

Lesion Type	Lesion diameter (mm)	Shape accuracy in ShearScan (%)	Shape accuracy in SSI (%)
4	4	89.8	68.0
	11	92.3	88.7
3	4	74.7	33.4
	11	79.5	85.5
2	4	61.7	60.4
	11	88.2	33.3
1	4	75.5	42.0
	11	89.7	61.1
Mean	81.4	59.0	

## 4 Discussion

Through our study, both 2D SWE systems showed very high intra- and inter-observer reproducibility (>0.9) in measuring elasticity within the lesion and the ratio between the lesion and background. In terms of intra- and inter-observer reproducibility, there have been a few reports about SE.^[16–18]^ Drakonaki et al^[16]^ studied the reproducibility of ultrasound elastography using a strain type elastography system (HV900, Hitachi Medical Corporation, Japan) by measuring a normal Achilles tendon and reported that the intra- and inter- values of the strain index for the transverse and longitudinal plane were 0.43, 0.45, 0.41, and 0.78, 0.66, 0.51, respectively. Another study^[17]^ using strain elastography in evaluating thyroid ultrasound showed 0.77 to 0.79 interobserver agreement and 0.73 to 0.87 intra observer reproducibility. Recently, Dong et al^[18]^ reported the inter-observer and intra-observer reproducibility of strain elastography in breast lesion, however, the results were poor to moderate (0.438, 0.365–0.655). On the other hand, the reports on the inter- or intra-observer reproducibility of SWE showed mostly excellent results.^[12,13,19]^ Our results also showed excellent results in both intra- and inter-observer reproducibility (0.990–0.999 and 0.991–1.000) with no significant difference between the 2 different shear wave systems of 2D SWE.

Liu et al^[17]^ reported that SWE showed comparable result to SE in diagnostic performance. Within the SWE system measuring shear wave speed, 2 systems were compared to evaluate the stiffness of the hepatic parenchyma, and ARFI (Siemens Medical Solutions, Erlangen, Germany) showed better intra- and inter-observer agreement than SSI.^[14]^ We could not evaluate diagnostic performance, but we could analyze the accuracy in measuring the known elasticity of the lesions within the elasticity phantom. In our study, the accuracy of Young's modulus was exceptionally high in both SSI and ShearScan (>0.9), but SSI showed lower accuracy (ICC 0.720–0.820) in measuring the elasticity in 4 mm size lesions compared with measuring 11 mm size lesions or compared with the results of ShearScan (*P* *<* .05). We cannot explain the exact reason, however, decreased accuracy in measuring the elasticity of 4 mm size lesion using SSI could have relation with the decreased shape accuracy in 4 mm size lesions with SSI.

In our experience of clinical practice, anterior superimposed color overlay mapping relative to the true lesion was predominantly observed in SSI, especially in very stiff lesions. Sometimes, color mapping is displayed just on the anterior side of the lesions and no color signal is observed within the lesion area. This inaccurate shape matching is confined to the SSI system rather than being a general characteristic of the elastography system, and considered as a system-specific error. Generally, accurate shape matching between the area of true lesion on B-mode image and color mapping of elastography is not important in differentiating benign and malignant lesion, especially when we measure the elasticity at the stiffest area within or around the lesion.

The accuracy of shape-matching on elastography image was significantly lower in SSI than ShearScan (81.4% vs 59.0%, respectively), and the poor shape accuracy was exaggerated in 4 mm lesion. Small lesions (4 mm) with low elasticity (type 2) showed just 33.3% of shape accuracy in SSI, but the shape accuracy was not correlated with the level of elasticity. ShearScan showed high shape accuracy in type 1 and 4 lesions while SSI showed high shape accuracy in type 3 and 4 lesions. Considering that the accuracy of measured elasticity was excellent except in 4 mm lesion with SSI, the result of our study suggests that the measurement of elasticity in small lesions might be influenced by the inaccurate shape matching. However, we could not explain the mechanism of poor accuracy of SSI in the small lesion. There is no report on the shape accuracy of 2D elastography. Further investigation is needed for the correlation between the shape accuracy and the accuracy of measurement of elasticity.

Our study was limited by the fact that we used a phantom as a study model and that in vivo studies with real breast lesions may not produce the same results as our analysis. Not only is the geometry off (either cylinders vs spheres) from real breast lesions, as well as material properties, also the phantom background is much more homogeneous, so phantom image interpretation might be much simpler leading to overly optimistic result and would represent an unrealistic upper limit on what would be expected in vivo. However, a phantom model is useful for studies that are difficult to realize in real life, such as comparing the exact elasticity of lesions in our study, as well as studies about comparing radiation doses to the patients^[20]^. By using modeled lesions for which the exact values of stiffness are known, we could accurately compare targeted properties of each elastography system. All lesions were located at the same depth within the phantom and the background surrounding the lesions was homogeneous and the same, so we could eliminate other factors that could effect on the results. The other limitation was that we compared 2 systems from many 2D SWE systems, and the results from other systems can be different. Further studies comparing multiple 2D SWE systems in clinical setting with real breast lesions surrounded by heterogeneous parenchyma should be followed to support our study results.

In conclusion, we can follow up breast lesions using both of the 2D SWE systems with little difference. Both SSI and ShearScan demonstrated excellent intra- and inter-observer reproducibility without significant difference. The accuracy of Young's modulus was high with both SSI and ShearScan. However, we should consider that SSI showed lower accuracy of Young's modulus and poor shape-matching between B-mode and elastography color image in small lesions less than 5 mm.

## Acknowledgments

The authors would like to thank Kiwan Choi, MA, and Hyoung-Ki Lee, PhD from Samsung Electronics for the technical assistance in measuring shape accuracy of 2D shear wave elastography.

## Author contributions

**Conceptualization:** Eun Young Ko.

**Data curation:** Jung Han Woo, Boo-Kyung Han.

**Formal analysis:** Eun Young Ko.

**Investigation:** Jung Han Woo, Eun Young Ko.

**Methodology:** Eun Young Ko.

**Resources:** Boo-Kyung Han.

**Supervision:** Eun Young Ko, Boo-Kyung Han.

**Writing – original draft:** Jung Han Woo.

**Writing – review & editing:** Eun Young Ko.
